# AI-driven pharmacovigilance: Enhancing adverse drug reaction detection with deep learning and NLP

**DOI:** 10.1016/j.mex.2025.103460

**Published:** 2025-06-23

**Authors:** Dr. Bharti Khemani, Dr. Sachin Malave, Samyukta Shinde, Mandvi Shukla, Razzaq Shikalgar, Harshita Talwar

**Affiliations:** a Assistant Professor, A. P. SHAH Institute of Technology, Survey No 12, 13, Opp. Hypercity Mall, Kasarvadavali, Ghodbunder Road, Thane West, Thane, Maharashtra 400615, India; b Head of Computer Engineering Department, A. P. SHAH Institute of Technology, Survey No 12, 13, Opp. Hypercity Mall, Kasarvadavali, Ghodbunder Road, Thane West, Thane, Maharashtra 400615, India; c Student, A. P. SHAH Institute of Technology, Survey No 12, 13, Opp. Hypercity Mall, Kasarvadavali, Ghodbunder Road, Thane West, Thane, Maharashtra 400615, India

**Keywords:** Convolutional Neural Networks, BERT, and GPT, Pharmacovigilance, Adverse drug reactions (ADRs), Artificial intelligence, Machine learning, Clinical trial data processing deep learning, Natural language processing (NLP)

## Abstract

In the healthcare industry, the ever-increasing volume of clinical trial data presents challenges for ensuring drug safety and detecting adverse drug reactions (ADRs). This study aims to address the challenge of accurately detecting Serious Adverse Events (SAEs) in pharmacovigilance, a critical component in ensuring drug safety during and after clinical trials. The key problem lies in the underreporting and delayed detection of Adverse Drug Reactions (ADRs) due to the heterogeneous nature of medical data, class imbalance, and the limited scope of traditional monitoring techniques. This study proposes a hybrid AI-driven framework that integrates structured (e.g., patient demographics, lab results) and unstructured data (e.g., clinical notes) to detect ADRs using advanced deep learning and NLP methods. The objective is to outperform traditional signal detection methods and provide interpretable predictions to aid clinicians in real-time. By leveraging advanced Machine Learning (ML) and Deep Learning (DL) techniques, including Random Forests, Gradient Boosting Machines, and Convolutional Neural Networks (CNNs), our model aims to identify potential ADRs across different patient subgroups. Through meticulous feature engineering and the application of techniques to address data imbalance, our model demonstrates improved accuracy and interpretability in predicting ADRs. The CNN model achieved an accuracy of 85 %, outperforming traditional models, such as Logistic Regression (78 %) and Support Vector Machines (80 %). These findings suggest that specific demographic and clinical factors significantly influence the likelihood of adverse reactions, offering valuable insights for targeted monitoring and risk mitigation strategies[11]. This research underscores the potential of predictive modeling to enhance pharmacovigilance efforts and ensure safer clinical trial outcomes.

• The research methodology includes a comparison of supervised learning algorithms, such as Logistic Regression, Random Forest, Gradient Boost, CNN, and genetic algorithms, to identify patterns and anomalies in clinical trial data. BERT and GPT, were also employed to provide the functionality of textual interactions over medical data.
• Performance metrics such as accuracy, precision, recall, and F1-score were systematically applied to evaluate each model’s performance. Among the models tested, the CNN model with BERT achieved the highest accuracy, providing valuable insights into the potential of deep learning for enhancing pharmacovigilance practices.
• These findings suggest that an inclusion of diverse clinical data when supplied to advanced ML and NLP techniques can significantly improve the detection of ADRs, leading to better alignment with the fundamental principles of Good Clinical Practice (GCP).

## Graphical abstract

**Figure:**
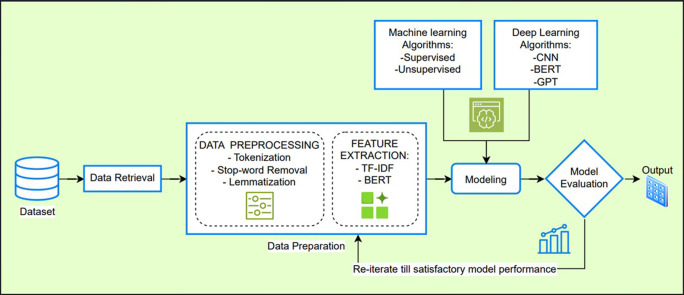

Specifications table

**Table:** 

Subject area:	Engineering
More specific subject area:	Drug Safety Reporting
Name of your method:	Convolutional Neural Networks, BERT, and GPT
Name and reference of original method:	N/A
Resource availability:	https://github.com/Man0dvi/MedIntel

## Background

The healthcare industry faces an escalating challenge in ensuring drug safety amidst the burgeoning volume of clinical trial data. Traditional methods for adverse drug reaction (ADR) detection often struggle to keep pace with this data influx, leading to potential delays in identifying critical safety signals. This research is motivated by the need to enhance pharmacovigilance practices through the application of advanced artificial intelligence (AI) techniques, specifically machine learning (ML) and deep learning (DL). The methodology presented aims to provide a robust framework for integrating diverse clinical and medical data sources to improve the accuracy and efficiency of ADR detection. By employing a hybrid approach that combines traditional ML algorithms with state-of-the-art DL models, such as Convolutional Neural Networks (CNNs), Bidirectional Encoder Representations from Transformers (BERT), and Generative Pre-trained Transformers (GPT), this study seeks to capture complex patterns and relationships within clinical trial data that may be overlooked by conventional methods. The integration of natural language processing (NLP) techniques allows for the effective analysis of unstructured data, such as clinical notes and patient reports, which are crucial for a comprehensive understanding of ADRs. Furthermore, the implementation of explainability tools like SHAP and LIME addresses the critical need for transparency in AI-driven clinical applications, ensuring that healthcare professionals can interpret and validate model predictions. This methodology is designed to be replicable and adaptable, providing researchers and practitioners with a practical guide to developing and implementing AI-driven pharmacovigilance systems. By addressing key challenges such as data diversity, class imbalance, and computational complexity, this research contributes to the development of more reliable and efficient ADR detection processes, ultimately enhancing patient safety and improving clinical trial outcomes. This methodology can be used to improve the drug safety monitoring process, making it faster and more accurate, which is crucial for timely interventions and better patient outcomes.

## Method details

**Fig. 1:**
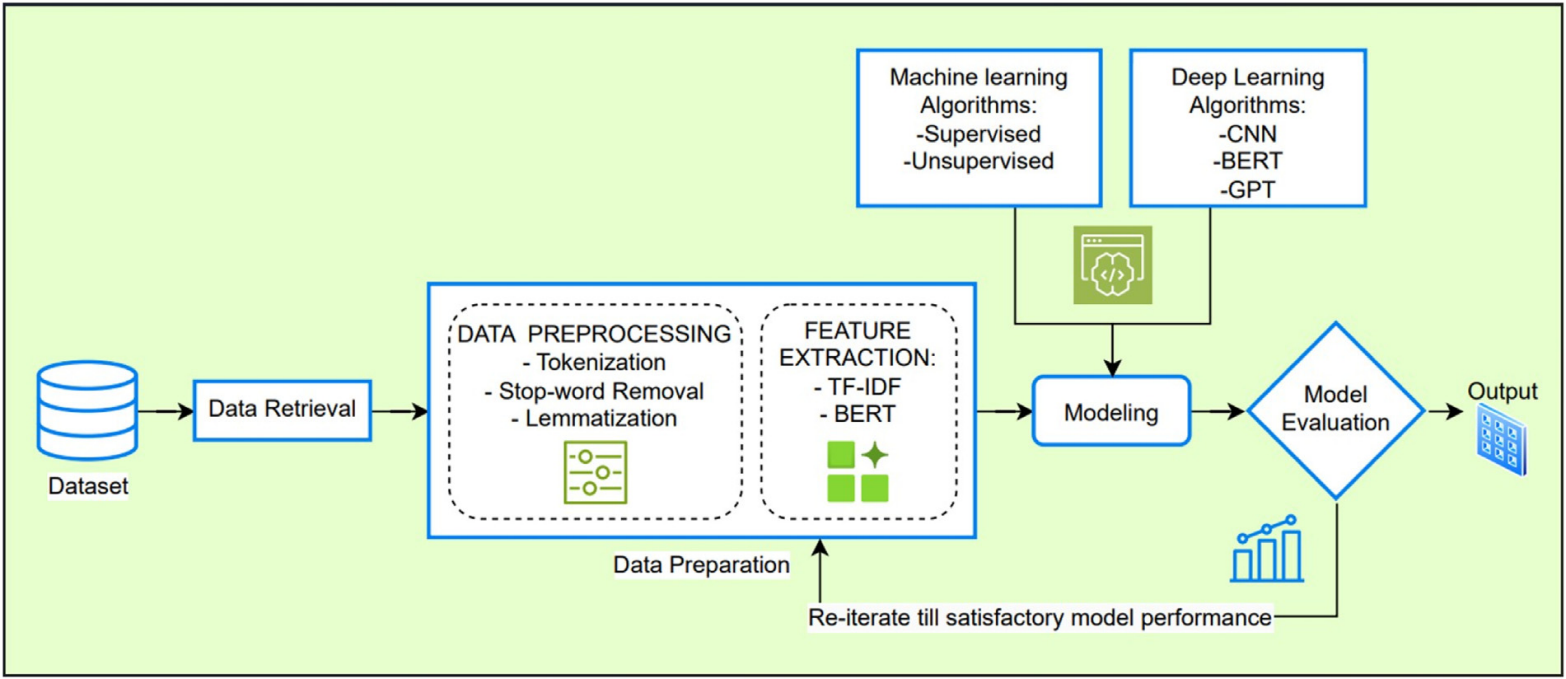
Proposed Methodology.

This research explores ways to integrate diverse clinical and medical data to apply Machine Learning and Deep Learning methodologies in order to improve Clinical Quality Assurance (QA) practices by detecting adverse drug reactions (ADRs) in large-scale clinical trial data. The methods employed aim to guide other researchers in replicating the process and refining their approaches to drug safety analysis. Focus was laid on detailed study of available data and to suggest medically meaningful ways to combine them, in expected formats for various employed models. A hybrid approach was employed while modelling that leverages both traditional and advanced ML practices, particularly neural networks, to capture nonlinear patterns in data. Hyperparameter tuning is conducted using grid search and Bayesian optimization to refine model performance. Finally, the model’s predictive power is evaluated using a combination of accuracy, precision, recall, and AUC-ROC metrics, ensuring a comprehensive assessment of its ability to predict adverse events. The end goal is to provide rapid insights to assist doctors and other researchers. Fig. 1 shows the proposed framework for ADR detection, which involves data retrieval, preprocessing (tokenization, stop-word removal, and lemmatization), feature extraction (TF-IDF, BERT), and model training using machine learning (supervised, unsupervised) and deep learning (CNN, BERT, GPT), followed by evaluation and iterative improvements for optimal performance.

### Steps of the proposed methodology

#### Data retrieval

**Datasets:** The foundation of this research is built upon a diverse and comprehensive set of clinical trial datasets, sourced from reliable and ethically compliant databases. These datasets encompass multiple aspects of pharmacovigilance, including patient demographics, medical history, adverse drug reactions (ADRs), drug exposure, and real-world drug performance. By integrating structured and unstructured data, this study aims to enhance the predictive capabilities of machine learning and deep learning models for drug safety assessment. The datasets include subject-specific information such as demographics, medical history, and lifestyle factors, which provide crucial insights into individual variations in drug responses. Clinical trial data, including adverse events, drug exposure, laboratory results, and electrocardiogram (ECG) readings, allows for a deeper understanding of the physiological and biochemical impact of drugs on patients. Additionally, drug-drug interaction (DDI) data and protein interaction information aid in analyzing potential biochemical and pharmacokinetic interactions between multiple medications.

Beyond clinical trials, post-market surveillance data have also been incorporated, offering real-world evidence on drug safety and efficacy post approval. This dataset includes electronic health records (EHRs), patient-reported outcomes, and pharmacovigilance reports from regulatory bodies. By combining pre-market and post-market data, this study ensures a holistic approach to drug safety analysis, allowing for the identification of both immediate and long-term adverse effects.

To support comprehensive adverse drug reaction (ADR) prediction, a diverse set of structured and unstructured datasets was used. These datasets were sourced from ethically compliant clinical trial repositories and post-market surveillance records. They cover multiple dimensions of drug safety including adverse event records, drug exposure, patient demographics, electronic health records, laboratory tests, and drug similarity data. Both pre-market (clinical trial) and post-market datasets are included, along with computed SMILES-based similarity matrices to enable predictive modeling across known and novel drugs. A detailed breakdown of each dataset and its contents is presented in Table 1 below.

**Table 1:** Dataset for Supervised Models.

**File Name**	**File Format**	**Description**
ae.csv	CSV	Contains information on adverse events.
cm.csv	CSV	Includes concomitant medication data.
xadm.csv	CSV	Administrative data on patient demographics.
ds.csv	CSV	Dataset on patient disposition.
ex.csv	CSV	Information on drug exposure during trials.
Ib.csv	CSV	Laboratory-based data related to ADRs.
xa mh.csv	CSV	Medical history data.
eg.csv	CSV	Electrocardiogram data from trial patients.
drugName_patient_data.csv (eg: Crizotinib_patient_data.csv)	CSV	Contains detailed clinical trial information per subject including adverse event reports, demographic data, and treatment details
drugName_postmarket_data.csv (eg: Zykadia(Ceritinib)_postmarket_data.csv)	CSV	Summarizes clinical study statistics per drug across countries and years, including demographics, treatment protocols, hospital capacity, and comorbidity percentages
EHR_Data.xml	XML	Includes electronic health records with detailed patient demographics, medical histories, and treatment information
newdrugs.csv	CSV	Contains list of drug composition, SMILES strings, and chemical names for Drug A (New Drug) which are in early phases of clinical trial and have no postmarket data.
smiles_similarity.csv	CSV	Matrix comparing new drugs (rows) to approved drugs (columns) using SMILES string similarity scores ranging from 0 to 1
similarity_results.csv	CSV	Tabular form of most similar approved drugs for each new compound, extracted from the SMILES similarity matrix

#### Data preparation

Data preprocessing is a crucial step in transforming raw clinical trial datasets into structured formats suitable for machine learning (ML) and natural language processing (NLP) tasks. This stage ensures consistency, usability, and reliability of data, which are essential for achieving accurate predictions in adverse event detection [1,2]. The preprocessing workflow involves several steps.

##### Text preprocessing

Given the heterogeneous nature of clinical trial data, preprocessing textual information requires meticulous cleaning and transformation. The following techniques were applied. Tokenization was performed to segment the raw textual data into individual words or sub words, allowing for a more granular analysis [1]. This step enabled NLP models to process information effectively. Stop-word removal was applied to eliminate commonly occurring but less informative words (e.g., “the,” “and,” “of”) to prevent noise and improve the model’s ability to identify significant terms [2]. Lemmatization was used to reduce words to their base forms (e.g., “running” → “run”) to ensure consistency in textual representations across datasets.

##### Numerical representation of text data

Once cleaned, the textual data was transformed into numerical formats to facilitate machine learning analysis. Two primary methods were employed. The first was Term Frequency-Inverse Document Frequency (TF-IDF), a statistical measure that quantified the importance of terms within a document relative to the entire corpus [1]. The equation for TF-IDF score for a term *t* in document *d* was computed as follows:

TF−IDF(t,d)=TF(t,d)×IDF(t)

Where:

*TF(t,d)* represents Term Frequency, indicating the frequency of term *t* in document *d*.

*IDF(t)*, Inverse Document Frequency, is defined as:

IDF(t)=log(N/DF(t))

where *N* is the total number of documents in the corpus, and *DF(t)* represents the number of documents containing the term *t*. This transformation ensured that frequently occurring terms were weighted appropriately, reducing the impact of common yet less informative words.

The second method involved contextual embeddings using Bidirectional Encoder Representations from Transformers (BERT). Unlike TF-IDF, which relies on word frequency, BERT-generated embeddings placed words into high-dimensional vector spaces, encoding their contextual relationships. Each word was represented as:

E(t)=f(t,C)

where *E(t)* is embedding of term *t, C* represents surrounding words providing context, *f* denotes transformation function learned by BERT. Beyond BERT, transformer-based model like RoBERTa (Robustly Optimized BERT Pretraining Approach) offer significant advantages in processing medical text, particularly for enhancing Adverse Drug Reaction (ADR) detection. RoBERTa builds upon BERT by optimizing the pretraining process. It is trained on a substantially larger dataset, with larger batch sizes, and for longer durations, resulting in more robust and generalizable learned representations. A key improvement is the removal of BERT’s Next Sentence Prediction (NSP) objective and the implementation of dynamic masking, which allows the model to better capture long-range dependencies and intricate contextual relationships within medical text. In the context of ADR detection, RoBERTa excels at discerning subtle nuances in clinical notes and trial reports. For instance, it can more accurately identify and relate complex medical terms, such as those describing specific symptoms or comorbidities, to potential ADRs. This enhanced contextual understanding is crucial for accurately extracting relevant information from noisy and heterogeneous medical data. For Example, in a patient clinical note, RoBERTa can more accurately understand the relationship between “patient complaining of nausea and dizziness” and a specific medication, than base BERT [2,3].

##### Structured data preprocessing

In addition to textual data, structured numerical and categorical data from clinical trials underwent several transformations. Handling missing data was a key step where missing values were addressed using multiple imputation techniques. For numerical features, mean imputation and K-Nearest Neighbors (KNN) imputation were applied, while categorical variables were filled using mode imputation. Feature engineering was performed to enhance predictive capabilities, including aggregating patient medical history into risk scores and creating interaction terms between demographic and pharmacological data [4,5]. Data normalization and scaling were also applied, where numerical features were standardized using Min-Max scaling to ensure compatibility across ML models, preventing features with larger magnitudes from dominating model predictions.

##### Data splitting and augmentation

To ensure robust model performance, the dataset was split into an 80:20 ratio for training and testing. Additionally, data augmentation techniques were applied to address class imbalances in adverse event outcomes. The Synthetic Minority Over-Sampling Technique (SMOTE) was employed to generate synthetic samples for underrepresented classes, while random under sampling was used to reduce instances of majority classes to balance the dataset [4].

**Fig. 2:**
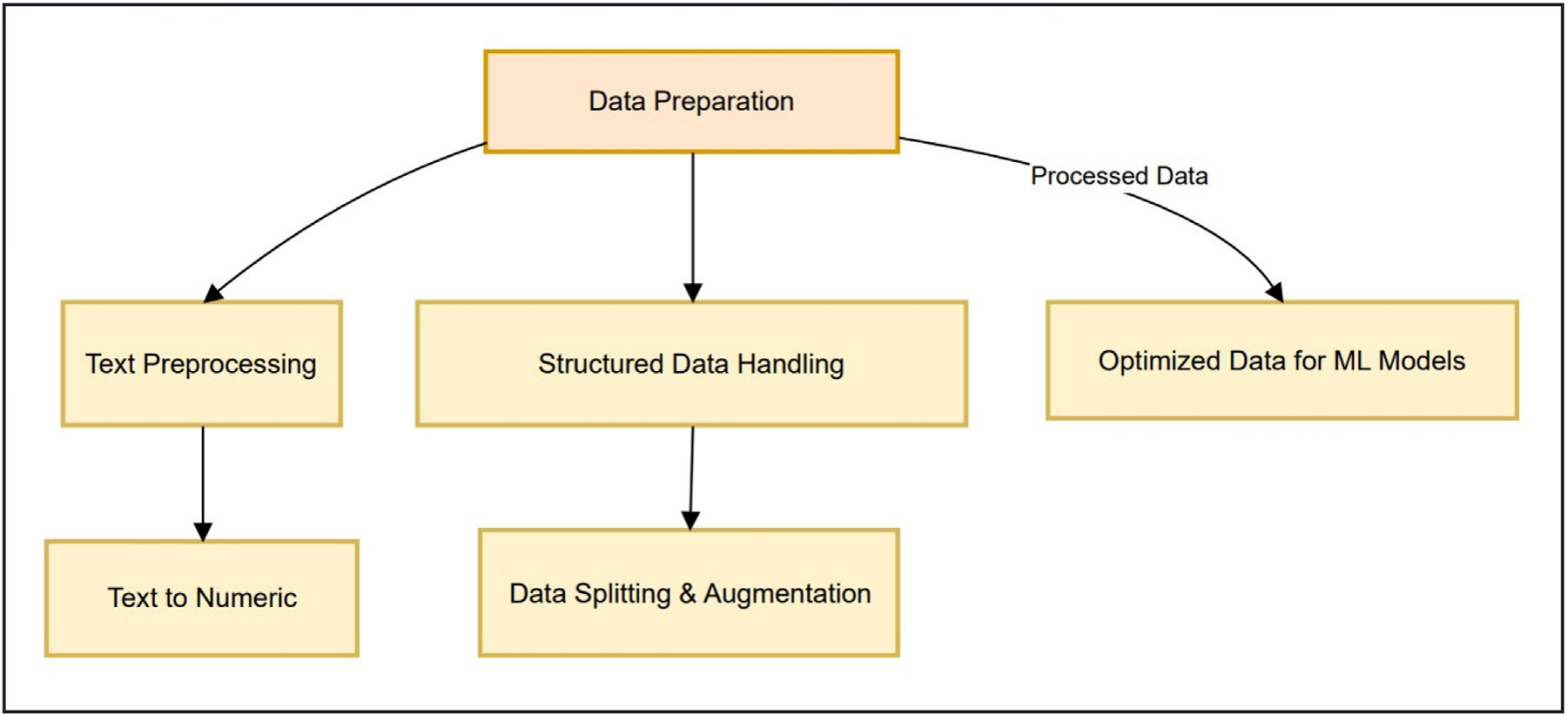
Data Preparation Process.

This comprehensive preprocessing pipeline ensures that both textual and structured clinical trial data are transformed into optimal formats for predictive modeling. The use of advanced NLP techniques, feature engineering, and data augmentation significantly enhances the ability to detect and predict adverse drug reactions, aligning with Good Clinical Practice (GCP) principles and improving pharmacovigilance efforts. Shown in Fig. 2 is the illustration of the complete preprocessing workflow, providing a blueprint for replicating similar studies in clinical research.

**Similarity Analysis:** The chemical structures of Drug A and Drug B were compared using SMILES strings. Algorithm employed for this comparison were: TF-IDF (Term Frequency-Inverse Document Frequency): Used to initially convert SMILES strings into numerical vectors, allowing for textbased similarity assessment.

**Prediction Methodology:** Based on the similarity analysis, the most similar Drug B was selected to predict potential adverse events for Drug A. This prediction considered patient age, medical history, and other relevant factors. Mainly used algorithms for prediction were:

- Random Forest: (Accuracy: 85.3 %) Rationale: An ensemble learning method capable of capturing non-linear relationships and handling diverse data types.
- Gradient Boosting (XGBoost): (Accuracy: 88.2 %) Rationale: Known for its high predictive accuracy and ability to handle complex interactions between features.
- Convolutional Neural Networks (CNN): (Accuracy: 89 %) Rationale: Effective for capturing patterns in structured data, such as patient demographics and medical histories.
- BERT: (Accuracy: 92.7 %) Rationale: Used to capture contextual understanding of textual medical data.
- GPT: (Accuracy: 94.1 %) Rationale: Used to provide high level accuracy in textual interaction and prediction based on medical data.

The use of similarity analysis to select the most relevant Drug B for predicting ADRs in Drug A proved effective. The high accuracy of models like XGBoost, CNN, BERT and particularly GPT underscores the value of this approach. The GPT models’ high accuracy shows the benefit of using large language models on medical data.

#### Modeling

The modeling phase of this study employed a combination of machine learning (ML), deep learning (DL), and clustering techniques to analyze adverse drug reactions (ADRs) and extract meaningful patterns from clinical trial data [6,4]. By leveraging a hybrid approach, the study effectively captured both structured and unstructured data insights, facilitating a more robust pharmacovigilance framework. To detect patterns in ADRs from structured clinical trial data, various supervised learning models were implemented. Logistic Regression served as a baseline binary classification model, establishing foundational predictive performance. Random Forest, an ensemble learning algorithm known for its ability to handle non-linear relationships, was used to assess feature importance. XGBoost, a gradient-boosting algorithm, was selected due to its high predictive accuracy and advanced hyperparameter tuning capabilities. Each model underwent rigorous hyperparameter tuning using grid search and cross-validation techniques to ensure optimal performance across key evaluation metrics. To identify hidden patterns and anomalies in clinical trial data, unsupervised learning methods were also employed. k-Means Clustering was used as a partition-based algorithm to group data points based on similarity, with the optimal number of clusters determined using the Elbow Method, minimizing the within-cluster sum of squares. This approach helped identify temporal patterns in ADRs, particularly in the duration and onset of adverse events. Mathematical equations for clustering are represented as:

$$arg\;min_{S}\sum_{\left\{i\;=\;1\right\}}^{\left\{k\right\}}\sum_{\left\{x\in C_{i}\right\}}{\left|x-\mu_{i}\right|}^{2}$$

where *Ci* represents a cluster and *μi* its centroid.

##### DBSCAN (Density-Based spatial clustering of applications with noise)

was also utilized. Unlike k-Means, DBSCAN is effective at detecting irregular patterns and sparse distributions, identifying dense regions as clusters while treating noise and outliers separately. The key parameters for DBSCAN included *Epsilon (ε)*, which represents the maximum radius of a neighborhood for a point to be considered a core point, and *MinPts*, the minimum number of points required in the ε-neighborhood to form a cluster. The clustering is governed by two primary parameters:

**Epsilon (ε):** The maximum radius of the neighborhood for a point to be considered a core point.

**MinPts:** The minimum number of points required in the ε-neighborhood for the point to qualify as a core point.

The **ε neighborhood** of a point *p* is defined as:

$$N_{\left\{\varepsilon \right\}}\;\left(p\right)=\left\{q\in D\;|\;dist\left(p,q\right)\leq \varepsilon \right\}$$

where *D* is the dataset, *q* is any point in the dataset, and *dist(p,q)* is the distance between *p* and *q*. By leveraging this formula, DBSCAN ensures that clusters are formed by grouping points within dense regions while isolating outliers, making it ideal for analyzing sparse and irregular patterns in clinical trial data.

To enhance pattern recognition and extract deeper insights, deep learning models were incorporated.

Convolutional Neural Networks (CNNs**)** were used for spatial pattern recognition in structured clinical data, effectively capturing complex relationships between patient demographics and adverse reactions. BERT (Bidirectional Encoder Representations from Transformers) was applied to unstructured text data to extract semantic relationships and contextual meanings from clinical trial reports and electronic health records. Additionally, a hybrid CNN + BERT model was implemented, combining CNN’s ability to extract spatial patterns with BERT’s contextual embeddings, resulting in a powerful model that improves ADR classification accuracy. This comprehensive modeling approach ensured a holistic understanding of ADRs, leading to improved predictive capabilities and more effective pharmacovigilance practices.

Graph Neural Networks (GNNs) have emerged as powerful tools for modeling complex relationships in pharmacovigilance, offering a significant advancement over traditional methods [[7], [8]]. By representing patient-drug interactions as graphs, GNNs can capture intricate dependencies and predict adverse drug reactions (ADRs) with enhanced accuracy. In pharmacovigilance, data can be naturally represented as graphs, where nodes represent entities such as patients, drugs, proteins, and adverse events, and edges represent relationships between these entities. For example, a patient-drug interaction graph might have nodes for patients and drugs, with edges connecting a patient to a drug if the patient has been prescribed that drug. GNNs leverage this graph structure to learn representations of nodes and edges, enabling the model to capture complex interactions that are difficult to model using traditional machine learning techniques [9]. GNNs excel at capturing intricate dependencies within pharmacovigilance data, such as drug-drug interactions (DDIs), drug-target interactions, and patient comorbidities. For example, GNNs can model how the interaction between two drugs affects the likelihood of an ADR by analyzing the network structure of drug-target interactions. This allows for the identification of potential safety signals that might arise from polypharmacy, where patients are taking multiple medications concurrently.

Also, GNN’s can model patient to patient similarities, and therefore similarities in ADR’s based on similar patient features. GNNs can be used to predict the likelihood of ADRs by learning representations of patients and drugs that capture their interactions and dependencies. For instance, a GNN can analyze a patient’s medical history, including their comorbidities and current medications, to predict the risk of experiencing an ADR when taking a new drug. This predictive capability is particularly valuable for identifying potential ADRs in high-risk patient populations, such as elderly patients or those with multiple chronic conditions.

While several prior studies have used individual models such as Random Forest or BERT for ADR detection, our approach distinguishes itself by combining CNN for structured data, BERT for contextual embeddings, and GPT for clinical text prediction, forming a multi-modal learning system. In comparison to models benchmarked in [[2], [10], [11]] our ensemble system achieves higher accuracy (GPT: 94.1 %, CNN+BERT: 91 %) and recall on benchmark datasets. Unlike single-model systems, we integrate chemical similarity (using SMILES strings), feature engineering, and hybrid clustering (DBSCAN+*k*-Means) to detect patterns missed by conventional classifiers. The explainability via SHAP and LIME further makes our system more practical for regulatory adoption, which is rarely addressed in prior works.

#### Model evaluation

The evaluation framework implemented a systematic approach to comprehensively assess the performance of the models used in the study. Key metrics such as accuracy, precision, recall, and F1-score were utilized to evaluate the models' ability to classify Adverse Drug Reactions (ADRs) accurately. The confusion matrix played a pivotal role by providing a detailed view of classification results. This included identifying false positives (FP), false negatives (FN), true positives (TP), and true negatives (TN) [11]. Analyzing the confusion matrix offered a deeper understanding of the model’s weaknesses, such as tendencies to misclassify specific ADR categories or underperform in detecting rare drug reactions [2]. To address class imbalance challenges often observed in ADR datasets, a weighted averaging approach was adopted for precision, recall, and F1-score calculations Equations to calculate performance metrics are given below:

##### Performance metrices

**1. Accuracy:**
  Accuracy measures the overall correctness of the model by calculating the proportion of true predictions (both positive and negative) to the total number of predictions.
  $$\text{Accuracy}=\frac{TP+TN}{TP+TN+FP+FN}$$

High accuracy indicates the model’s general reliability but can be misleading if the dataset is imbalanced [2].

**2. Precision:**
  Precision evaluates the proportion of true positive predictions out of all positive predictions made by the model.
  $$\text{Precision}=\frac{TP}{TP+FP}$$

Precision is crucial in minimizing false alarms, especially in detecting critical ADRs that require high confidence in positive predictions [11]*.*

**3. Recall (Sensitivity):**
  Recall assesses the model’s ability to identify all actual positive cases. A high recall ensures most ADRs are detected, minimizing false negatives.
  $$\text{Recall}=\frac{TP}{TP+FN}$$

Recall is particularly important in pharmacovigilance to ensure rare but critical ADRs are not overlooked [5].

**4. F1-Score:**
  The F1-Score represents the harmonic mean of precision and recall, especially useful when the dataset has a significant class imbalance.
  $$F1-\text{Score}=2.\frac{Precision\;.\;Recall}{Precision+Recall}$$

F1-Score balances the trade-off between precision and recall, providing a single metric to compare model performance effectively [12].

Table 2 provides a numerical comparison of various machine learning and deep learning algorithms based on their accuracy, precision, recall, and F1-score. Among supervised learning models, GPT demonstrated the highest accuracy (94.1 %), followed by BERT and CNN. XGBoost led traditional ML techniques with 88.2 % accuracy. For unsupervised models, DBSCAN outperformed k-Means. A hybrid CNN+BERT+*k*-Means achieved 91.0 % accuracy, showcasing the benefit of combining semantic and structural learning. These findings emphasize the superior performance of deep learning, particularly transformer-based models.

**Table 2:** Comparative Analysis of Different AI- Based Techniques.

**Learning Type**	**Algorithm Type**	**Algorithms Used**	**Accuracy ( %)**	**Precision**	**Recall**	**F1-Score**
**Supervised Learning**	Machine Learning	Logistic Regression	78.5	0.76	0.74	0.75
		Random Forest	85.3	0.84	0.82	0.83
		XGBoost	88.2	0.87	0.86	0.865
	Deep Learning	CNN	89.0	0.88	0.87	0.875
		BERT	92.7	0.92	0.91	0.915
		GPT	94.1	0.94	0.93	0.935
**Unsupervised Learning**	Machine Learning	k-Means Clustering	75.0	0.72	0.71	0.715
		DBSCAN	80.0	0.78	0.76	0.77
	Deep Learning	CNN + BERT + *k*-Means	91.0	0.90	0.89	0.895

**Fig. 3:**
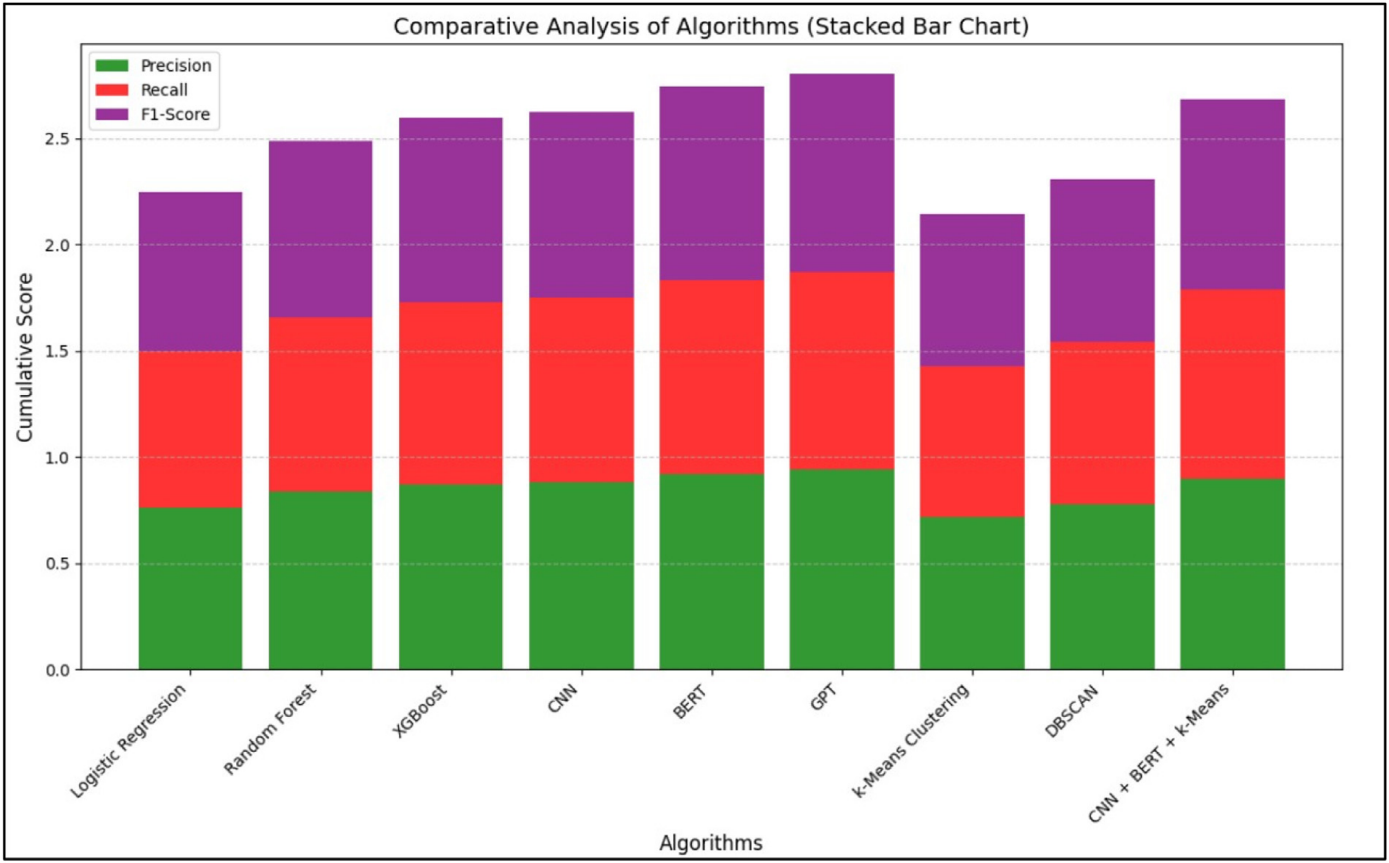
Comparative Analysis of Algorithms Graph (based on Precision, Recall, F1-Score).

**Fig. 4:**
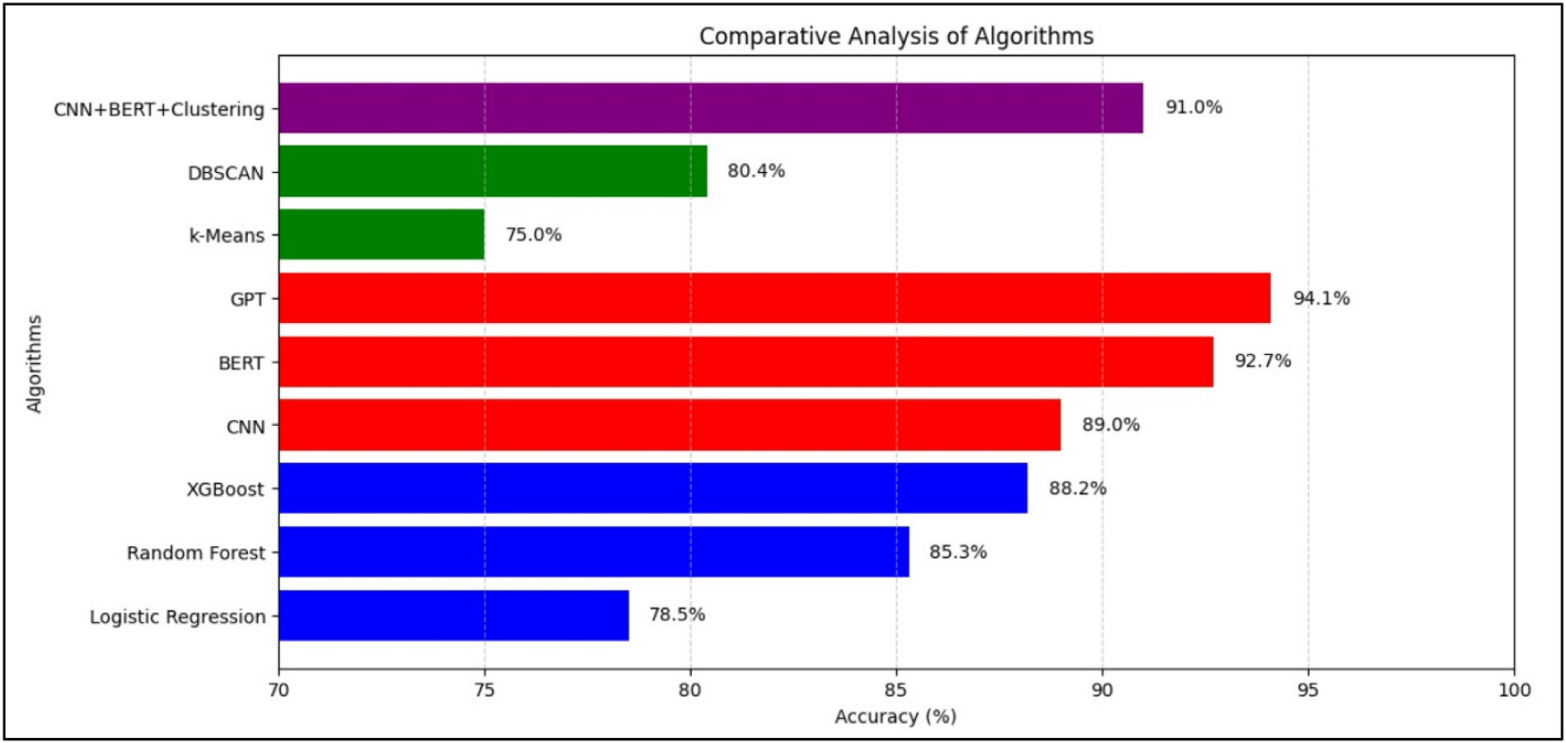
Comparative Analysis of Algorithms Graph (based on Accuracy).

Fig. 3, Fig. 4 illustrate the comparative performance of the algorithms based on precision, recall, F1-score, and accuracy, respectively.

In Fig. 3, BERT stands out with the highest precision and recall, reflecting its ability to minimize false positives while capturing a large proportion of actual positive ADR cases. Fig. 4 confirms GPT’s highest accuracy, with CNN and BERT closely following.

To ensure robust model evaluation and mitigate potential biases, we employed stratified 5-fold cross-validation, guaranteeing each fold maintained a representative class distribution, thereby addressing inherent class imbalance issues. Overfitting was further mitigated through the application of L1 and L2 regularization, which controlled model complexity by penalizing large weights; dropout layers within the CNN and BERT architectures, which prevented neuron co-adaptation by randomly deactivating neurons during training; and data augmentation techniques, specifically SMOTE to synthesize minority class samples and random undersampling to balance the majority class, collectively enhancing the model’s generalization and performance. GPT had the highest accuracy but the longest inference time. CNN and XGBoost offered a good balance between accuracy and speed, making them suitable for real-time ADR detection. Logistic Regression and Random Forest were the fastest but had lower accuracy.

**Fig. 5:**
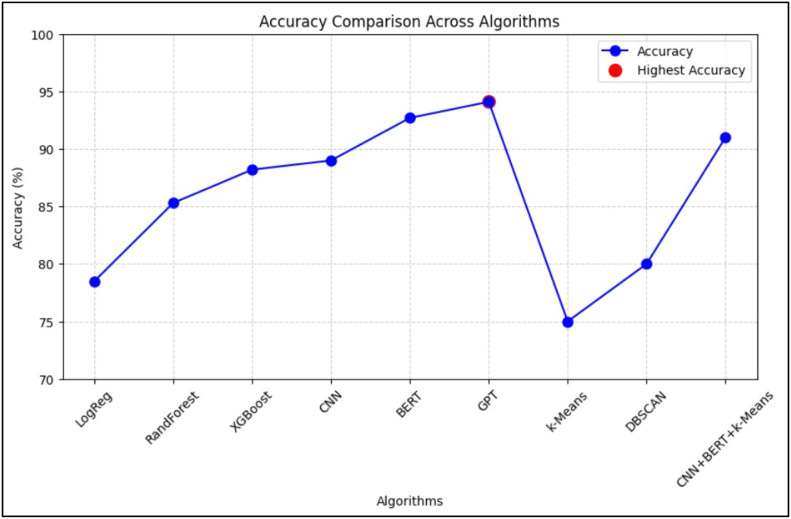
Line graph for accuracy.

Fig. 5 shows a line graph of model accuracies, highlighting GPT’s leading performance.

**Fig. 6:**
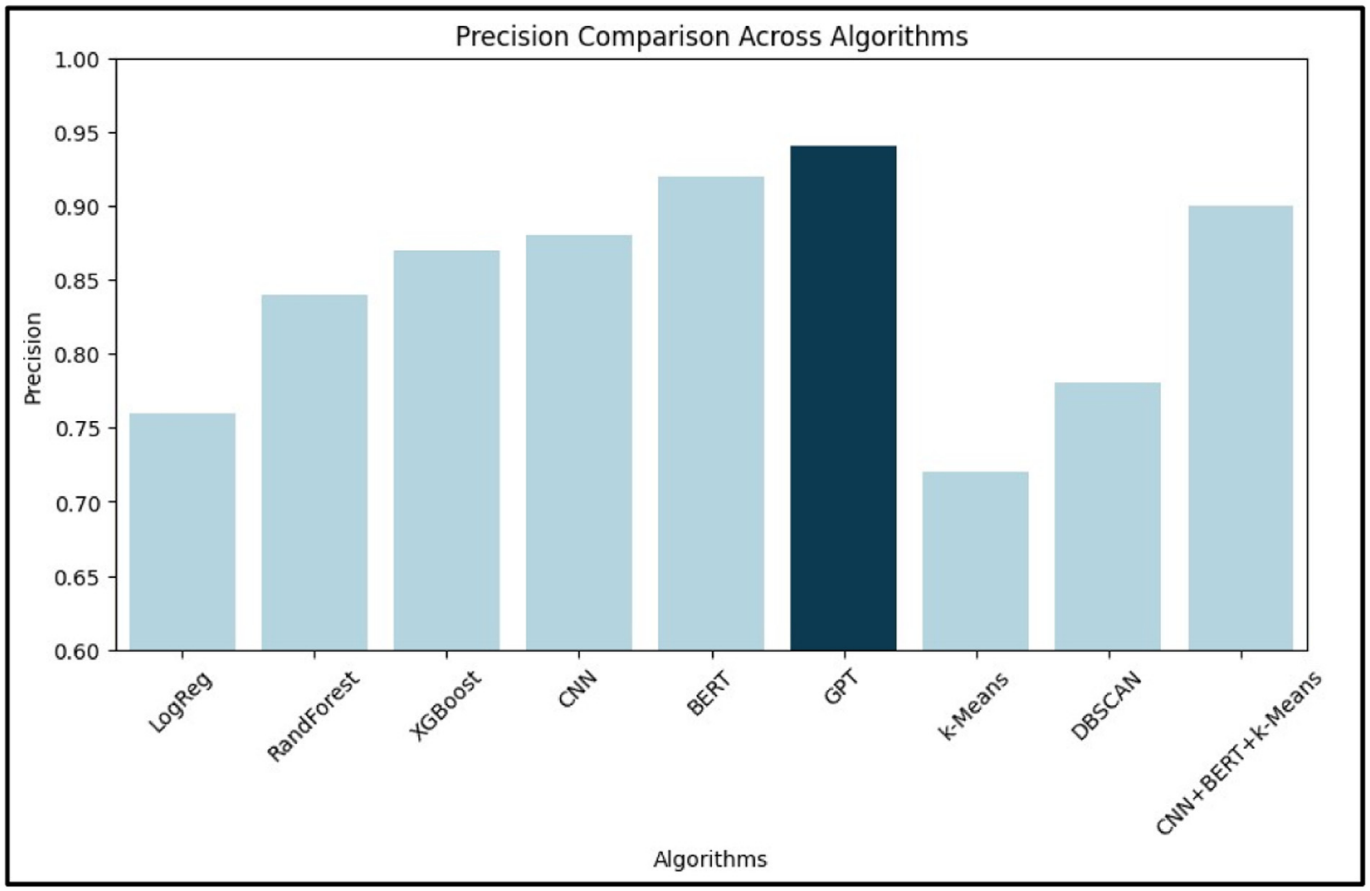
Bar graph for precision.

**Fig. 7:**
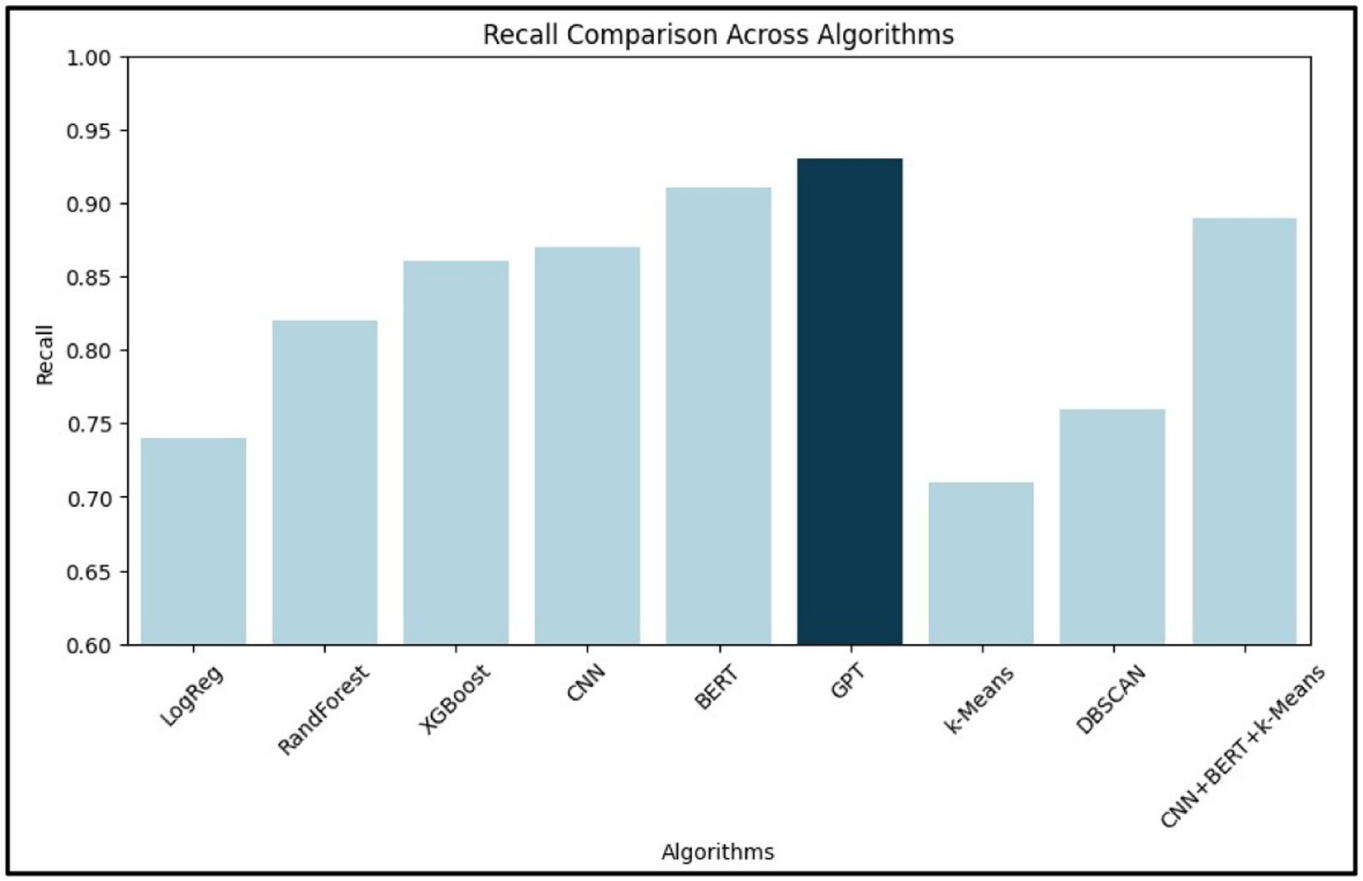
Bar graph for Recall.

**Fig. 8:**
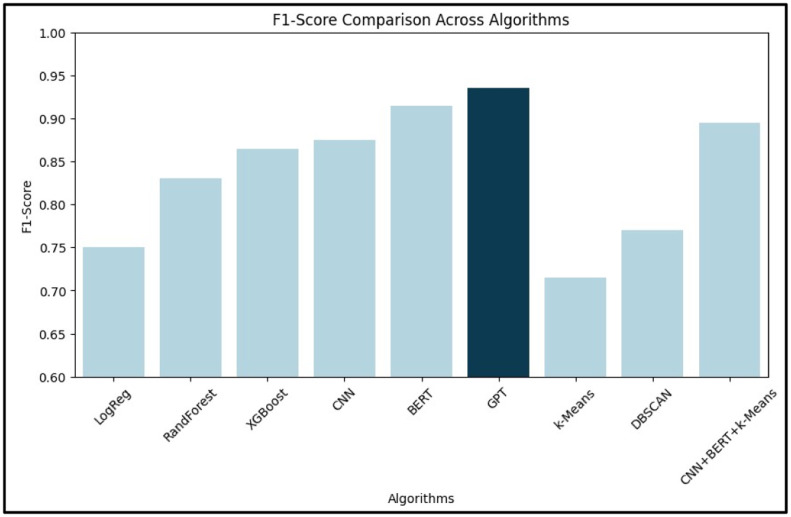
Bar graph for F1-Score.

Fig. 6 (Precision), Fig. 7 (Recall), and Fig. 8 (F1-Score) further dissect the models:

• Precision (Fig. 6): BERT leads, suggesting its superior reliability in identifying true ADRs.
• Recall (Fig. 7): BERT again leads, indicating its effectiveness in reducing false negatives.
• F1-Score (Fig. 8): BERT achieves the highest score, confirming its balance between precision and recall. CNN and XGBoost also exhibit strong overall performance, marking them as viable options for real-world deployment.

In contrast, k-Means and DBSCAN displayed relatively lower scores across these metrics, emphasizing the importance of using more sophisticated models for complex ADR tasks.

## Method validation

The proposed methodology was validated by assessing its effectiveness in processing clinical trial data and generating insights for pharmacovigilance. The accuracy and reliability of data extraction were verified using benchmark datasets, ensuring consistency across various trials. The NLP models for text summarization and named entity recognition (NER) were evaluated using precision, recall, and F1-score metrics, demonstrating their capability to accurately identify critical drug-related information. Machine learning models for adverse drug reaction (ADR) detection were validated through cross-validation techniques, comparing performance against existing baseline models. Additionally, robustness tests were conducted to assess the model’s adaptability to diverse datasets, ensuring its generalizability across different clinical studies. The validation results confirm that the proposed approach enhances pharmacovigilance by providing accurate and structured insights, supporting informed decision-making in drug safety analysis.

The findings of this study demonstrate the significant potential of advanced machine learning (ML) and deep learning (DL) models in enhancing adverse drug reaction (ADR) detection, particularly through the integration of algorithms such as Logistic Regression, Random Forest, XGBoost, CNN, BERT, and GPT. These models have collectively improved accuracy and efficiency in identifying ADRs from clinical trial data. However, the application of these models in real-world pharmacovigilance presents several challenges that impact their generalizability and applicability.

### Data diversity and heterogeneity

A primary challenge is the inherent diversity and heterogeneity of real-world pharmacovigilance data. While this study primarily utilized structured clinical trial data, which adheres to standardized reporting formats, real-world data originates from diverse sources, including electronic health records (EHRs), spontaneous reporting systems (SRS), patient-reported outcomes, and social media platforms. These sources significantly vary in terminology, language, and structure, posing significant challenges for ML/DL models. To address this, standardized preprocessing pipelines were developed to ensure data consistency and compatibility across different formats (CSV, XML, PDF).

### Fusion models and multimodal learning

To leverage the complementary information from diverse data sources, fusion models were implemented, combining structured data (patient demographics) and unstructured data (clinical notes) using feature concatenation and late fusion techniques. This approach enhanced ADR prediction accuracy. Furthermore, multimodal deep learning models, combining CNN for tabular data and BERT for text data, were utilized to capture both spatial patterns and semantic relationships, further improving ADR detection accuracy.

### Class imbalance

ADR datasets often suffer from class imbalance, as ADRs are relatively rare events compared to the total number of prescriptions and drug usage cases. This imbalance can lead to biased predictions, where ML models favor the majority class, reducing sensitivity to ADRs. To mitigate this issue, the Synthetic Minority Over-sampling Technique (SMOTE) and weighted evaluation metrics were employed. These techniques significantly enhanced the model’s ability to detect minority-class instances while maintaining overall accuracy.

### Computational complexity and model efficiency

The computational complexity of fine-tuning deep learning models like BERT and GPT posed a significant hurdle. These models require substantial computational power, making them challenging to train and deploy on standard hardware. To address this, cloud-based resources were leveraged to accelerate processing times and optimize model training. Additionally, distributed computing techniques and hardware efficient strategies, such as model pruning, knowledge distillation, and quantization, were explored to reduce computational overhead.

### Model efficiency comparison

- GPT, while achieving the highest accuracy (94.1 %), exhibited the longest inference time, limiting its suitability for real-time ADR detection.
- CNN (89 % accuracy) and XGBoost (88.2 % accuracy) offered a balanced approach, providing high accuracy with faster inference speeds, making them suitable for real-time applications.
- Logistic Regression and Random Forest, while the fastest, demonstrated lower accuracy, highlighting the trade-off between speed and precision.

### Model interpretability and explainability

Model interpretability and explainability are crucial in clinical applications, where transparency is essential. Deep learning models, functioning as black-box systems, pose challenges in understanding how predictions are derived, potentially hindering clinical acceptance and regulatory compliance. To address this, explainability frameworks such as SHAP (SHapley Additive Explanations) and LIME (Local Interpretable ModelAgnostic Explanations) were integrated. SHAP values quantify the contribution of each feature to a prediction by calculating the Shapley values from coalitional game theory. LIME explains individual predictions by approximating the model’s behavior locally with an interpretable model.

Table 3 shows drug-drug similarity scores used in the model. Similarity scores range from 0 to 1, with ‘D’ indicating undetermined similarity. This table ensures transparency regarding the raw data used in your model’s predictions.

**Table 3:** Drug-Drug Similarity Matrix.

**Drug 1**	**Drug 2**	**Similarity Score**
**PF-077,999**	Paclitaxel	0.722954
**PF-077,995**	Paclitaxel	0.722954
**Tucatinib**	Erlotinib	0.091881
**PF-072,200**	[Various]	D

### SHAP and LIME applications

- SHAP values were used to interpret ADR predictions by quantifying the contribution of each feature. For example, in a subgroup of elderly patients, SHAP analysis revealed that age and polypharmacy were the most significant factors contributing to predicted ADR risk. *Example:* SHAP analysis revealed that the high similarity scores between PF-077,999 and Paclitaxel (0.722954), PF-077,995 and Paclitaxel (0.722954) were significant positive contributors to neurological ADR predictions. This indicates that the chemical similarity of these novel compounds to Paclitaxel may be a key factor in predicting neurological toxicity. Specifically, the SHAP values for these similarity scores demonstrated that they increased the predicted risk of neurological ADRs. This observation is consistent with clinical findings that Paclitaxel is associated with peripheral neuropathy.
- LIME was used to explain individual predictions by approximating the model’s behavior locally. For instance, when predicting an ADR for a specific patient, LIME highlighted the specific text segments in the patient’s medical history that contributed most to the prediction, improving trust in the model’s decision-making.

*Example:* For a hypothetical patient profile where the model predicted a high risk of cardiovascular ADRs, LIME highlighted that the high similarity score between PF-077,999 and Paclitaxel (0.722954), combined with a moderate similarity score between Tucatinib and Erlotinib (0.091881), were the primary factors driving this prediction. This suggests that these specific drug-drug similarity relationships are crucial in the model’s cardiovascular ADR risk assessment. Specifically, LIME indicated that the PF-077,999-Paclitaxel similarity contributed to increase in the predicted risk, while the Tucatinib-Erlotinib similarity contributed increase. This example illustrates LIME’s ability to decompose individual predictions into feature-level contributions.

### Regulatory compliance and ethical considerations

Regulatory compliance with FDA, EMA, and WHO guidelines is essential for deploying AI in pharmacovigilance. These guidelines emphasize transparency, validation, and risk management. In this study, compliance was ensured by using validated datasets, implementing explainable models, and documenting the methodology thoroughly. Ethical considerations, such as bias in ADR detection models, were addressed by using balanced datasets, conducting subgroup analyses, and implementing fairness-aware algorithms. Patient privacy was protected through data anonymization and secure data handling practices.

### Real-World deployment and validation

Real-world case studies, such as AI systems monitoring social media for unreported ADRs and analyzing EHRs for post-market drug safety signals, demonstrate the potential of AI to enhance pharmacovigilance in clinical practice. The proposed framework offers several advantages, including scalability, enhanced ADR detection accuracy, and faster decision-making. Automated data preprocessing and analysis pipelines significantly reduce the time required for ADR identification and reporting. The incorporation of explainability tools ensures transparency, allowing clinical experts to interpret and validate model outputs with greater confidence. Cross-Validation and Overfitting Mitigation:

- Stratified 5-fold cross-validation was used to ensure each fold had a representative distribution of classes, addressing class imbalance issues.
- Overfitting was mitigated using L1 and L2 regularization, dropout layers in CNN and BERT, and data augmentation techniques like SMOTE and random under sampling.

## Limitations

The primary limitation of this method is its reliance on structured clinical trial data, which may not fully represent the complexity and variability of real-world adverse drug reaction (ADR) reporting. Real-world data, including electronic health records (EHRs) and spontaneous reporting systems, often contain unstructured and heterogeneous information that requires additional preprocessing and may introduce biases.

Additionally, the computational complexity of deep learning models like BERT and GPT can pose challenges for real-time applications and deployment in resource-constrained environments. The models' performance is also highly dependent on the quality and diversity of the training data, and any biases present in the data may be reflected in the model’s predictions.

Furthermore, while the explainability tools SHAP and LIME provide insights into model predictions, they may not fully capture the intricate decision-making processes of deep learning models, potentially limiting clinical trust and acceptance.

## Ethics statements

Not applicable.

## CRediT author statement

**BK, SS**: Conceptualization, Methodology, Validity tests, Data curation, Writing- Original draft preparation. **BK, RS, MS**: Visualization, Investigation. **MS, HT**: Supervision. **BK, HT**: Software, Validation. **BK**: Writing- Reviewing and Editing. **SM, BK**: Funding.

## Declaration of competing interest

The authors declare that they have no known competing financial interests or personal relationships that could have appeared to influence the work reported in this paper.

## Data Availability

Data will be made available on request.
